# White matter hyperintensities associated with progression of cerebral small vessel disease: a 7-year Chinese urban community study

**DOI:** 10.18632/aging.103154

**Published:** 2020-05-10

**Authors:** Yiwei Xia, Yi Shen, Yi Wang, Lumeng Yang, Yiqing Wang, Yu Li, Xiaoniu Liang, Qianhua Zhao, Jianjun Wu, Shuguang Chu, Zonghui Liang, Xiaoxiao Wang, Bensheng Qiu, Hansheng Ding, Ding Ding, Xin Cheng, Qiang Dong

**Affiliations:** 1 Department of Neurology, National Clinical Research Centre for Aging and Medicine, Huashan Hospital, State Key Laboratory of Medical Neurobiology, Fudan University, Shanghai, China; 2 Institute of Neurology, National Clinical Research Centre for Aging and Medicine, Huashan Hospital, Fudan University, Shanghai, China; 3 Centers for Biomedical Engineering, University of Science and Technology of China, Hefei, Anhui, China; 4 Department of Neurology, Jing’an District Center Hospital, Shanghai, China; 5 Department of Radiology, Shanghai East Hospital, Tongji University School of Medicine, Shanghai, China; 6 Department of Radiology, Jing’an District Center Hospital, Shanghai, China; 7 Shanghai Health Development Research Center (Shanghai Medical Information Center), Shanghai, China

**Keywords:** white matter hyperintensities, lacunes, cerebral microbleeds, enlarged perivascular spaces, cognition

## Abstract

We aimed to explore the role of white matter hyperintensities (WMH) in progression of cerebral small vessel disease (CSVD) in an urban community in China over a period of 7 years, and to investigate associations between WMH volume (baseline and progression) and cognitive impairment. CSVD markers and neuropsychological tests at baseline and follow-up of 191 participants of the Shanghai Aging Study (SAS) were assessed. WMH volume were assessed by automatic segmentation based on U-net model. Lacunes, cerebral microbleeds (CMBs) and enlarged perivascular spaces (ePVS) were rated manually. Small vessel disease (SVD) score was rated as the total burden of CSVD markers. Global cognitive function and 5 main cognitive domains (memory, language, spatial construction, attention and executive function) were evaluated by neuropsychological tests. We performed multivariable linear regression and binominal logistic regression. Participants with higher baseline WMH volume developed more progression of WMH volume, increased risk of incident lacunes, incident CMBs, and ePVS progression. WMH (baseline and progression) were associated with decline of executive function. WMH were associated with progression of cerebral small vessel disease and decline of executive function in a Chinese urban community study over a period of 7 years.

## INTRODUCTION

Brain imaging studies in the general population have demonstrated that markers of cerebral small vessel disease (CSVD), including white matter hyperintensities (WMH), lacunes, cerebral microbleeds (CMBs), and enlarged perivascular spaces (ePVS), are highly prevalent in individuals over 60 years of age [1–4]. CSVD markers, and their progression, have been recognized as important vascular contributors to cognitive impairment and dementia [5, 6]. Therefore, investigation on progression of these markers is crucial to better understanding of both etiology and consequences of CSVD.

WMH is the most common marker among these CSVD markers, detected in approximately 90% of individuals older than 60 years of age in the general population [7]. Several longitudinal community studies indicated that high burden of WMH at baseline was associated with progression of WMH [8–11], incident CMBs [2, 12] and progression of ePVS [13]. However, the association between baseline WMH and incident lacunes remains uncertain, though one study validated this association in a hospital cohort [14].

Since WMH are associated with the progression of other markers, WMH might be an early precursor in the progression of CSVD. However, one study found that baseline ePVS was significantly associated with an increased risk of lacunes, CMBs, and progression of WMH, suggesting that ePVS might be an early precursor [13]. Therefore, investigation on the role of baseline WMH in the progression of all these markers would help to disentangle the complex interplay between these markers.

Current knowledge regarding progression of CSVD in China is limited due to lack of longitudinal studies based on Chinese community. Two cross-sectional studies [15, 16] in rural regions of China described the prevalence of CSVD markers, but neither of them provided data on progression of CSVD.

In this longitudinal study, we systematically explored the associations between baseline WMH volume and progression of all these CSVD markers in an urban community in China over a period of 7 years. In addition, we investigated the associations between WMH volume (baseline and progression) and cognitive impairment.

## RESULTS

Characteristics of the 191 participants at baseline and follow-up were presented in Table 1. Median age at baseline was 68.1 years and 43.5% were male. Mean follow-up duration was 6.9 + 0.4 years (minimum, 4.7 years; maximum, 8.0 years). Significant changes from baseline to follow-up were observed in CSVD markers including WMH volume, CMBs and extensive ePVS, SVD score, and cognitive function including MMSE, memory, spatial construction, attention and executive function. Those lost to follow-up were significantly older and had more baseline vascular risk factors, CSVD burden and worse cognitive function compared to those followed (Supplementary Table 1).

**Table 1:** Characteristics of the study population at baseline and follow-up (n=191).

	**Baseline (n=191)**	**Follow-up (n=191)**	**Change**	**P**
Demographics				
Age, y, median (IQR)	68.1(63 to 72.6)	74.6(69.6 to 79.3)	6.0 (6.0 to 7.0)	-
Sex, male, n (%)	83(43.5)	83(43.5)	-	-
Education, y, median (IQR)	12(9 to 15)	12(9 to 15)	-	-
Vascular risk factors				
Body mass index, kg/m^2^, meidan (IQR)	24.5(21.9 to 27.3)	23.4(21.6 to 26.1)	-1.0(-2.3 to 0.3)	**<0.001**
ApoE ε4 carriers, n (%)	26(14.0)	26(14.0)	-	**-**
Current smoking, n (%)	21(11.0)	15(7.9)	-6(3.1)	0.293
Hypertension, n (%)	90 (47.1)	107(56.0)	17(8.9)	0.082
Diabetes, n (%)	20(10.5)	28(14.7)	8(4.2)	0.217
Hyperlipidemia, n (%)	74(38.7)	85(44.5)	11(5.8)	0.254
Cardiogenic disease, n (%)	19(10.0)	28(14.7)	9(4.7)	0.161
Medication use, n (%)				
Antihypertensive	84(44.0)	106(55.5)	22(11.5)	**0.024**
Antidiabetic	17(8.9)	33(17.3)	16(8.4)	**0.015**
Lipid lowering	7(3.7)	36(18.9)	29(15.2)	**<0.001**
Antiplatelet / anticoagulation	33(17.3)	31(16.2)	-2(1.1)	0.784
CSVD markers				
WMH volume, %, meidan (IQR)	0.29(0.15 to 0.52)	0.59(0.29 to 0.92)	0.24 (0.07 to 0.45)	**<0.001**
Lacunes, n (%)	22 (11.5)	35 (18.3)	13 (6.8)	0.062
CMBs, n (%)^a^	16 (10.1)	53 (33.3)	37 (23.3)	**<0.001**
Extensive ePVS, n (%)	23 (12.0)	54 (28.3)	31 (16.2)	**<0.001**
SVD score≥2, n(%)^a^	17(10.7)	49(30.8)	32(20.1)	**<0.001**
Cognition, median(IQR)				
MMSE	29 (28 to 30)	29 (27 to 29)	-1(-2 to 0)	**<0.001**
Memory	0.31(-0.36 to 0.88)	-0.32(-0.89 to 0.23)	-0.61 (-1.24 to -0.02)	**<0.001**
Language	0.36 (-0.07 to 0.57)	0.25 (0 to 0.47)	0 (-0.22 to 0.22)	0.474
Spatial construction	0.11 (-0.61 to 0.83)	0 (-0.61 to 0.59)	-0.24 (-0.96 to 0.24)	**0.033**
Attention	-0.02(-0.54 to 0.60)	-0.34(-0.90 to 0.11)	-0.34 (-0.92 to 0.50)	**<0.001**
Executive function	0.07 (-0.09 to 0.23)	0.07(-0.25 to 0.23)	0 (-0.32 to 0)	**0.328**

^a^ For ratings of CMBs, 32 participants were additionally excluded based on missing T2*-GRE at baseline.

Abbreviations: CSVD = cerebral small vessel disease; WMH = white matter hyperintensities; CMBs = cerebral microbleeds; ePVS = enlarged perivascular spaces; SVD = small vessel disease; MMSE = Mini-Mental State Examination; IQR = interquartile range.

### Role of WMH in progression of CSVD

The associations between baseline CSVD markers and progression of each marker were demonstrated in Table 2. Participants with more baseline WMH volume developed more change of WMH volume (β=0.25, 95%CI 0.12-0.38), more risk of incident lacunes (OR 3.78, 95%CI 1.35-10.62), incident CMBs (OR 4.45, 95%CI 1.41-14.03), and ePVS progression (OR 2.98, 95%CI 1.22-7.28) (Model 1). When further adjusted for demographic factors, vascular risk factors and medication use (Model 2, Model 3, Model 4), all associations persisted. The associations did not change when measuring baseline WMH by Fazekas score (Supplementary Table 2). Higher baseline SVD score was associated with more change of WMH volume, more risk of incident lacunes and incident CMBs (Supplementary Table 3).

**Table 2:** Relationships between baseline CSVD markers and progression of CSVD markers.

	**Change of WMH (%)**	**Incident lacunes**	**Incident CMBs**	**ePVS Progression**
	β (95%CI)	OR (95%CI)	OR (95%CI)	OR (95%CI)
**Model 1**				
WMH volume, per 1% increase	**0.25(0.12,0.38)**	**3.78(1.35,10.62)**	**4.45(1.41,14.03)**	**2.98(1.22,7.28)**
Lacunes, per No. increase	**0.13(0.03,0.23)**	**3.13(1.42,6.88)**	1.33(0.67,2.65)	2.11(0.94,4.70)
CMBs, per No. increase	-0.00(-0.04,0.04)	0.64(0.28,1.45)	0.96(0.64,1.44)	0.83(0.59,1.18)
ePVS, per score increase	0.03(-0.05,0.10)	0.69(0.26,1.80)	0.95(0.56,1.63)	0.23(0.12,0.44)
**Model 2**				
WMH volume, per 1% increase	**0.25(0.13,0.38)**	**3.96(1.29,12.19)**	**4.10(1.29,13.09)**	**2.89(1.14,7.32)**
Lacunes, per No. increase	**0.11(0.01,0.21)**	**3.33(1.42,7.82)**	1.35(0.66,2.76)	2.30(1.00,5.29)
CMBs, per No. increase	0.01(-0.03,0.05)	0.60(0.21,1.70)	0.96(0.65,1.43)	0.81(0.57,1.15)
ePVS, per score increase	0.02(-0.05,0.09)	0.71(0.26,1.93)	0.95(0.55,1.64)	0.23(0.12,0.44)
**Model 3**				
WMH volume, per 1% increase	**0.24(0.11,0.37)**	**5.50(1.35,22.44)**	**4.60(1.39,15.23)**	**3.52(1.30,9.53)**
Lacunes, per No. increase	**0.13(0.03,0.24)**	**2.97(1.08,8.13)**	1.34(0.62,2.89)	2.18(0.90,5.31)
CMBs, per No. increase	0.00(-0.04,0.05)	0.66(0.24,1.82)	0.91(0.63,1.33)	0.81(0.56,1.19)
ePVS, per score increase	0.03(-0.04,0.10)	0.78(0.24,2.54)	0.78(0.43,1.42)	0.20(0.10,0.41)
**Model 4**				
WMH volume, per 1% increase	**0.23(0.10,0.36)**	**5.86(1.38,24.82)**	**4.76(1.38,16.44)**	**3.81(1.38,10.49)**
Lacunes, per No. increase	**0.13(0.03,0.23)**	**3.05(1.10,8.47)**	1.35(0.62,2.93)	2.28(0.93,5.60)
CMBs, per No. increase	0.01(-0.03,0.05)	0.65(0.25,1.71)	0.90(0.61,1.32)	0.84(0.57,1.23)
ePVS, per score increase	0.03 (-0.05,0.10)	0.77(0.24,2.54)	0.78(0.42,1.43)	0.17(0.08,0.37)

Model 1: unadjusted. Model 2: adjusted for baseline age, sex and interval. Model 3: adjusted for baseline age, sex, interval, BMI, ApoE ε4 carrier, current smoking, hypertension, diabetes, hyperlipidemia, cardiogenic disease. Model 4: additionally adjusted for antihypertensive, antidiabetic, lipid lowering, and antiplatelet / anticoagulation medications.

Abbreviations: CSVD = cerebral small vessel disease; WMH = white matter hyperintensities; CMBs = cerebral microbleeds; ePVS = enlarged perivascular spaces.

### Heterogeneity in progression of CSVD

**Figure 1:**
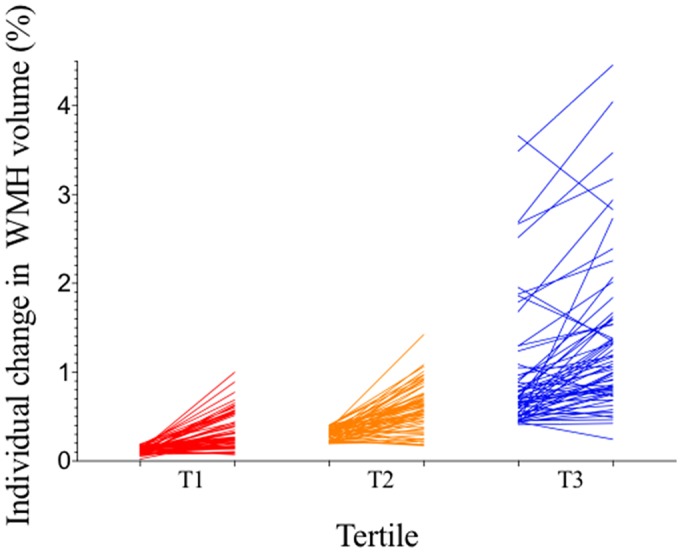
**Individual participants’ changes in WMH volume between baseline and follow-up by tertile of baseline WMH volume.** All participants (n=191) were divided into three groups by tertile of baseline WMH volume, with 64 participants in T1, 64 participants in T2, and 63 participants in T3. Each line represents an individual participant, linking baseline WMH volume (left) to follow-up WMH volume (right) of each tertile column. Participants in T1 had the least WMH progression and those in T3 had the most WMH progression. WMH = white matter hyperintensities.

**Figure 2:**
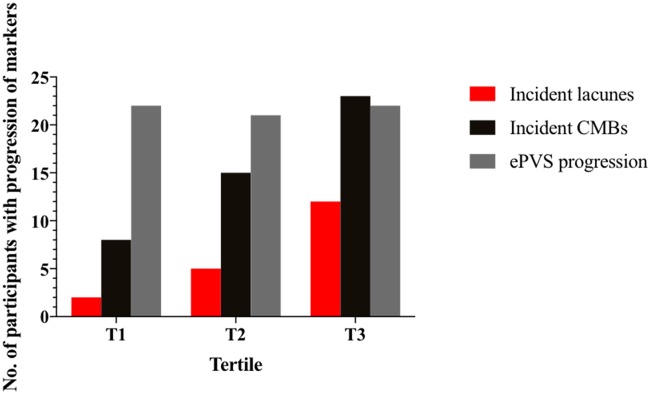
**Number of participants with progression of markers from baseline by tertile of baseline WMH volume.** Number of participants with incident lacunes, incident CMBs and ePVS progression were shown in columns by tertile of baseline WMH volume. Red column is the number of participants with incidents lacunes; black column is the number of participants with incidents CMBs; grey column is the number of participants with ePVS progression. Participants in T1 had the least progression of markers and those in T3 had the most progression of markers. CMBs = cerebral microbleeds; ePVS = enlarged perivascular spaces.

We plotted progression of CSVD markers by baseline WMH volume in tertile (Figures 1, 2). Median change of WMH volume over 7 years was 0.24% for all participants, 0.14% for participants with 1^st^ tertile WMH volume at baseline, 0.29% for those with 2^nd^ tertile WMH, and 0.30% for those with 3^rd^ tertile WMH. The heterogeneity in change of WMH was significant (P=0.024). Incident lacunes over 7 years developed in 19 of all 191 participants (9.9%). 2 of 64 (3.13%) with 1^st^ tertile WMH at baseline developed incident lacunes; 5 of 64 (7.81%) with 2^nd^ tertile WMH developed incident lacunes; 12 of 63 (19.0%) with 3^rd^ tertile WMH developed incident lacunes. The heterogeneity in incident lacunes was significant (P=0.011). Incident CMBs over 7 years developed in 46 of all 191participants (28.9%). 8 of 64 (12.5%) with 1^st^ tertile WMH at baseline developed incident CMBs; 15 of 64 (23.4%) with 2^nd^ tertile WMH developed incident CMBs; 23 of 63 (36.5%) with 3^rd^ tertile WMH developed incident CMBs. The heterogeneity in incident CMBs was significant (P=0.007). ePVS progression over 7 years developed in 65 of all participants (34.0%). 22 of 64 (34.4%) with 1^st^ tertile WMH at baseline had ePVS progression; 21 of 64 (32.8%) with 2^nd^ tertile WMH had ePVS progression; 22 of 63 (34.9%) with 3^rd^ tertile WMH had ePVS progression. The heterogeneity in ePVS progression was not significant (P=0.967).

### WMH and change of cognitive function

The relationships between CSVD markers (baseline and progression) and change of cognitive function were demonstrated in Tables 3 and 4, adjusted for age, sex, interval, education years and ApoE ε4 carriers. Higher burden of baseline WMH volume was associated with increased decline of MMSE (β=-0.95, P=0.036) and executive function (β=-0.45, P=0.004). Increased change of WMH volume was associated with increased decline of executive function (β=-0.50, P=0.010). In addition, increased ePVS progression was associated with increased decline of MMSE (β=-0.91, P=0.004). We did not observe any association between SVD score and change of cognitive function (Supplementary Tables 4, 5).

**Table 3:** Relationships between baseline CSVD markers and change of cognitive function.

	**Change of cognitive function**											
	**MMSE**		**Memory**		**Language**		**Spatial construction**		**Attention**		**Executive function**	
	**β**	**P**	**β**	**P**	**β**	**P**	**β**	**P**	**β**	**P**	**β**	**P**
WMH volume, per 1% increase	**-0.95**	**0.036**	0.41	0.054	0.02	0.918	0.51	0.092	-0.07	0.770	**-0.45**	**0.004**
Lacunes, per No. increase	-0.56	0.123	-0.20	0.235	0.23	0.153	0.11	0.651	0.47	0.009	-0.04	0.734
CMBs, per No. increase	0.18	0.249	-0.03	0.643	-0.07	0.299	-0.12	0.261	-0.10	0.215	0.07	0.168
ePVS, per score increase	0.34	0.185	-0.21	0.092	0.17	0.134	-0.09	0.597	-0.06	0.642	-0.06	0.478

Adjusted for age, sex, interval, education years, ApoE ε4 carrier

Abbreviations: CSVD= cerebral small vessel disease; WMH = white matter hyperintensities; CMBs = cerebral microbleeds; ePVS = enlarged perivascular spaces; MMSE = Mini-Mental State Examination.

**Table 4:** Relationships between progression of CSVD markers and change of cognitive function.

	**Change of cognitive function**											
	**MMSE**		**Memory**		**Language**		**Spatial construction**		**Attention**		**Executive function**	
	**β**	**P**	**β**	**P**	**β**	**P**	**β**	**P**	**β**	**P**	**β**	**P**
Change of WMH volume, per 1% increase	-0.11	0.842	0.28	0.290	-0.38	0.120	0.54	0.142	-0.29	0.297	**-0.50**	**0.010**
Incident lacunes, per No. increase	-0.12	0.594	-0.04	0.746	0.10	0.329	-0.03	0.831	-0.00	0.974	0.07	0.394
Incident CMBs, per No. increase	0.09	0.318	0.00	0.997	0.02	0.686	-0.01	0.918	0.01	0.874	-0.01	0.834
ePVS progression, per score increase	**-0.91**	**0.004**	-0.04	0.782	0.09	0.491	0.05	0.805	-0.01	0.928	-0.01	0.916

Adjusted for age, sex, interval, education years, ApoE ε4 carrier

Abbreviations: CSVD= cerebral small vessel disease; WMH = white matter hyperintensities; CMBs = cerebral microbleeds; ePVS = enlarged perivascular spaces; MMSE = Mini-Mental State Examination.

Forty-one (21.47%) participants developed change in cognitive diagnosis from baseline to follow-up. Among them, 36 (18.85%) participants had incident mild cognitive impairment (MCI); 4 (2.09%) participants had incident Alzheimer’s disease (AD); 1 (0.52%) participant had incident vascular dementia (VaD). We did not find any association between change in cognitive diagnosis and baseline CSVD markers or progression of CSVD markers (Supplementary Tables 6, 7).

## DISCUSSION

In this study over a period of 7 years in a Chinese cohort over 60 years, we found that WMH were associated with progression of CSVD and decline of executive function.

Our study demonstrated that baseline WMH were associated with incident lacunes in the general population in China, which helped to fill the gap of previous community studies. Additionally, baseline lacunes were associated with change of WMH, suggesting the close relationship between WMH and lacunes. One cross-sectional community study found that prevalent lacunes preferentially localized to the edge of white matter hyperintensities [17]. Another study on the etiology of incident lacunes hypothesized that WMH might convert to lacunes via an intermediate stage of a subtype of lacunes which is not cavitated yet [18]. These findings suggested potential shared pathophysiological mechanism of WMH and lacunes, and required to be further explored in future studies.

Some studies [19, 20] suggested that ePVS might be an early precursor of CSVD, but none of these studies systematically examined the associations between baseline markers and progression of them. One longitudinal study [13] observed the association between baseline ePVS and progression of CSVD markers, but the result is bidirectional, suggesting the complex temporality among these markers. In this Chinese urban community, baseline total SVD score (representing the total burden of CSVD) was associated with progression of only three markers, but baseline WMH was associated with progression of all four markers. These findings suggested WMH might play an important role in progression of CSVD and might be an early precursor of CSVD.

There might be a common underlying pathophysiological process resulting in the progression of all these markers. Blood-brain barrier (BBB) leakage hypothesis [21] pointed out that the loss of normal endothelial junction would result in leakage of plasma fluid components leading to rarefaction and demyelination of white matter (WMH), vessel wall thickening and luminal distortion eventually leading to secondary perforating arteriolar thrombosis, luminal occlusion and traditional ‘infarction’ (lacunes), tendency for microbleeds to cluster in the occipital lobes due to gravity (CMBs), and the failure of interstitial fluid drainage (ePVS). Further studies are required to explore the relationship between BBB permeability and progression of CSVD markers.

Executive function has long been considered a cognitive domain affected by vascular injuries [22]. Previous studies in US and European countries [23–25] indicated that CSVD markers were associated with executive function in the general population. In this study, we only found associations between WMH (baseline and progression) and decline of executive function in the Chinese population, suggesting the important role of WMH in predicting cognitive impairment.

Strengths of this study include the longitudinal study with long follow-up duration in an urban community in China, and comprehensive assessments of cognition. In addition, WMH were assessed quantitatively by automatic segmentation.

A limitation of our study is the variation of MRI scanners from baseline to follow-up. Since 3.0T scanners are more widely used these years, we used 3.0T scanner instead of 1.5T scanner at follow-up. Theoretically, 3.0T scanner could detect more lesions compared to 1.5T scanner, which might exaggerate the progression of CSVD markers. However, since all participants underwent the same MRI scans at the same time point, change of MRI resolutions could be regarded as a limited systematic error when examining the associations between baseline markers and progression of markers. Another limitation of our study is the attrition bias due to old age at baseline and long-term follow-up, probably leading to an underestimation of progression of CSVD, since those who dropped out were older and had more comorbidities. Among them, 6 participants with dementia were not able to cooperate in the follow-up examinations, inevitably leading to bias in the evaluation of cognitive impairment. However, the bias from these 6 participants was limited when considering the total sample size. Even in this relatively healthy cohort, we found baseline WMH were associated with progression of CSVD and decline of executive function.

## MATERIALS AND METHODS

### Study population

**Figure 3:**
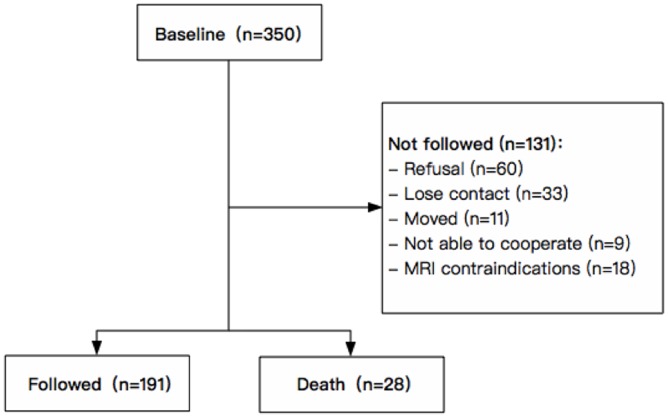
**Flowchart of recruitment for study subjects in Jing’an Temple Community.**

This study is part of the Shanghai Aging Study (SAS), which was a prospective, population-based cohort study in old people aged 60 or over in Jing’an Temple Community, an urban community in Shanghai, China. The detailed study protocol has been published previously [26]. 350 participants without dementia and stroke underwent baseline examination in 2009 to 2011. Potential participants were excluded if they had: (1) cerebral large vessel stenosis (>50%); (2) hydrocephalus or brain tumors; (3) MRI contraindications; (4) incorporative or not able to complete the examination. In 2016 to 2018, all eligible participants were invited for a second examination. Of the 350 participants at baseline, 28 people died, and 131 people were not eligible to participate in the second examination (60 refused, 33 could not be reached, 11 moved to other communities, 9 was not able to cooperate (6 had dementia and 3 had tumor), 18 had MRI contraindications). In total, 191 of the 350 eligible participants completed repeated examinations in 2016 to 2018 with a mean interval of 6.9 years (Figure 3).

### Ethic statements

This study was approved by the medical ethics committee of Huashan Hospital, Fudan University, Shanghai, China. Written informed consent was obtained from all participants or their legally acceptable representative.

### MRI acquisition

At baseline (2009~2011), participants underwent head MRI scans on a 1.5-Tesla GE scanner with following sequences: T1-weighed, T2-weighed, axial fluid-attenuated inversion recovery (FLAIR), T2*-weighted gradient recall echo (GRE), MRA sequence. Full acquisition details have been described previously [27].

At follow-up (2016~2018), repeated MRI scans on 3.0-Tesla GE scanner were conducted. The detailed parameters of sequences were as follows: 3D T1 BRAVO (flip angle 12 degrees, slice thickness 1.2mm), T2 PROPELLER (TR/TE = 7.586s/ 93.76ms, flip angle 140 degree, slice thickness 6.0mm, slice spacing 2.0mm), Cor CUBE FLAIR (TR/TE 6000/90ms, slice thickness 2.0mm), SWAN (TR/TE minimum/45ms, flip angle 15 degree, slice thickness 2.0mm), and MRA (TR/TE minimum/minimum, flip angle 20 degree, slice thickness 1.4mm).

### Evaluation of CSVD markers

CSVD markers were rated according to the Standards for Reporting Vascular Changes on Neuroimaging (STRIVE) criteria [4] by two certified and registered neurologists who were blinded to clinical data, and were ascertained by a senior neurologist in case of disagreements.

WMH volume were generated by an automatic WMH segmentation method based on U-net model [28]. The model was trained on 38 images on an 11GB GTX1080ti GPU (image sources: baseline and follow-up images of our study, and a public image data set of UMC Utrecht hospital [29]), using the axial slices of T2w FLAIR images as input images. Based on this model, segmentations of each image were generated automatically by 2.66 seconds. The automatic segmentations showed satisfactory correlations with manual segmentations of both baseline (r^2^=0.9717, p<0.001) and follow-up images (r^2^=0.9723, p<0.001) (Supplementary Figure 1). All WMH volume measurements were expressed as the percentage of that volume of the total intracranial volume (ICV), thereby adjusting for different head sizes. ICV was calculated by summing total brain volume, sulcal volume, and ventricular CSF volume. We additionally used Fazekas score (a semi-quantitative score ranging from 0 to 6) [30] for baseline WMH measurement.

Number of lacunes and CMBs were rated manually on FLAIR/T1/T2-weighted and T2*-GRE/SWAN scans according to the STRIVE criteria [4]. Incident lacunes or CMBs were defined as new lacunes or CMBs from baseline.

Severity of ePVS was assessed in basal ganglia (BG) and centrum semiovale(CS) respectively, on one slice and at one side in the most affected hemisphere only (0 = no ePVS, 1 = 1-10 ePVS, 2 = 11–20 ePVS, 3 = 21–40 ePVS, and 4 = 40 or more ePVS) according to Potter’s scale [31], creating total ePVS score ranging from 0 to 8. ePVS score≥2 in BG or CS was defined as extensive ePVS [32]. ePVS progression was considered as elevation of ePVS score from baseline.

### Total SVD score

Total SVD score was an ordinal scale representing the total burden of CSVD, expressed by the presence of each of the four MRI markers mentioned above. One point was awarded in case of periventricular WMH Fazekas score 3 (extending into the deep white matter), and/or in case of deep WMH Fazekas score 2 or 3 (confluent or early confluent). One point was awarded when one or more lacunes were present. One point was awarded when one or more CMBs were present. One point was awarded in case of ePVS score≥2 in BG or CS [33]. Total burden of CSVD of each participant was assessed by SVD score, resulting in a score ranging from 0 to 4.

### Cognitive function

Participants underwent neuropsychological tests at baseline and follow-up in the following domains: 1) global cognitive function: Mini-Mental State Examination (MMSE); 2) memory: Auditory Verbal Learning Test (AVLT) for participants with≥6 years of education, Huashan Object Memory Test (HOMT) for those with < 6 years of education; 3) language: Common Objects Sorting Test (COST); 4) spatial construction: Stick Test; 5) attention: Trail Making Test (TMT) for participants with≥6 years of education, Renminbi Test for those with < 6 years of education; 6) executive function: Conflicting Instructions Task.

Those neuropsychological tests were translated, adapted, and normed from Western countries based on the Chinese culture, and had been validated in previously published Chinese studies [34, 35]. Details of neuropsychological tests were described in Supplementary Materials. Z-score (individual test score minus mean baseline test score divided by the SD) was calculated for each cognitive domain. Change of cognitive function was expressed by change in MMSE score and change in Z-score of each cognitive domain from baseline.

We made cognitive diagnosis based on the neurologic, psychiatric, and neuropsychological data at baseline and follow-up. Mild cognitive impairment (MCI) was defined according to Petersen’s criteria [36]; Alzheimer’s disease (AD) was defined according to NINCDS-ADRDA criteria [37]; vascular dementia (VaD) was defined according to NINDS-AIREN criteria [38]. Change in cognitive diagnosis was defined as incident MCI, incident AD or incident VaD from baseline.

### Vascular risk factors and medication use

Demographic, vascular risk factors, and medication use were collected via an interviewer-administered questionnaire, and were further confirmed in patient history, which comprised age, sex, body mass index, ApoE ε4 carrier, current smoking, hypertension, diabetes, hyperlipidemia, cardiogenic disease (atrial fibrillation and coronary artery disease), antihypertensive, antidiabetic, lipid lowering and antiplatelet/anticoagulation medications.

### Data analysis

Statistical analysis was performed on Stata v14.0 (StataCorp, LLC). Median (interquartile range, IQR) was used to describe continuous variables because of the non-normal distribution. Number (percentage) was used to describe categorical variables. The Mann-Whitney U test or Kruskal Wallis test was used to compare continuous variables. The Chi-square test or Fisher’s exact test was used to compare categorical variables. The characteristics of the study population at baseline and follow-up were compared by Mann-Whitney U test in continuous variables, and Chi-square test or Fisher’s exact test in categorical variables.

Multivariable linear regression was used to examine the associations between change of WMH volume and baseline CSVD markers, baseline SVD score. Binominal logistic regression was used to examine the associations between progression of other three markers and baseline CSVD markers, baseline SVD score. Covariables were selected from univariable analyses and with known potential clinical significance based on previous studies [2, 8, 10, 12–14]. These covariables were adjusted in multivariable models. We further plotted progression of markers by baseline WMH volume in tertile using Prism 7 Graphpad (GraphPad Software, Inc). To evaluate the impact of CSVD markers and SVD score (baseline and progression) on change of cognitive function and change in cognitive diagnosis, we used multivariable linear regression and binominal logistic regression.

All coefficients (β), odds ratios, 95% confidence intervals and P values were estimated in a two-tailed manner. Differences were considered to be statistically significant at p<0.05.

## Supplementary Materials

Supplementary Methods

Supplementary Figure 1

Supplementary Tables
