# Supplementary material for: White matter hyperintensities associated with progression of cerebral small vessel disease: a 7-year Chinese urban community study
Source: Aging (Albany NY). 2020 May 10;12(9):8506–22. doi: 10.18632/aging.103154 (PMC7244059; doi:10.18632/aging.103154)
Supplement: Supplementary Methods [file aging-12-103154-s001.pdf]

SUPPLEMENTARY FIGURE

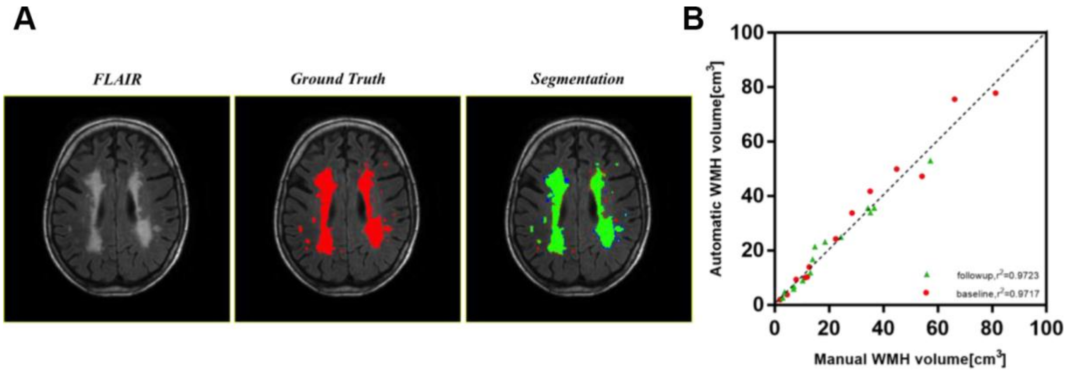

Supplementary Figure 1. Validation of automatic segmentation. (**A**) Manual and automatic segmentation of one case: original T2w FLAIR image, manual segmentation of WMH (red), and automatic segmention of WMH (green and blue). Green area represented the overlap between manual and automatic segmentation. (**B**) Pearson`s correlation between manual and automatic segmentation of 27 cases. Red dots represented baseline WMH volume, and green triangles represented follow-up WMH volume. Dashed line represented a 1:1 relation. WMH = white matter hyperintensities.
