# Supplementary material for: White matter hyperintensities associated with progression of cerebral small vessel disease: a 7-year Chinese urban community study
Source: Aging (Albany NY). 2020 May 10;12(9):8506–22. doi: 10.18632/aging.103154 (PMC7244059; doi:10.18632/aging.103154)
Supplement: Supplementary Figure 1 [file aging-12-103154-s002.pdf]

SUPPLEMENTARY METHODS

Semi-quantitative measurements for WMH

Since the quantitative measurement of WMH is more sensitive than those semi-quantitative measurements of other markers and might result in a bias in the conclusion, we additionally used a semi-quantitative method for baseline WMH measurement, i.e. Fazekas score. Periventricular WMH was graded as 0 = absence, 1 = “caps” or pencil-thin lining, 2 = smooth “halo”, 3 = irregular PVH extending into the deep white matter. Deep WMH was rated as 0 = absence, 1 = punctate foci, 2 = beginning confluence of foci, 3 = large confluent areas. A total Fazekas score ranging from 0 to 6 was generated [1]. We found WMH was still the only marker associated with the progression of all CSVD markers even when measuring WMH by Fazekas score (Supplementary Table 2).

Description of neuropsychological tests

Based on the Chinese culture, we translated, adapted, and normed neuropsychological tests from Western countries. Because some tests require vocabulary, writing, or reading skills, we designed the neuropsychological battery according to the education level of each participant. The battery was administered in Chinese by certified study psychometrists within 90 minutes. Neuropsychological tests and domains are as follows:

1. MMSE was used for screening for global cognition [2].

2. The Auditory Verbal Learning Test (AVLT), adapted from the California Verbal Learning Test, was used to measure verbal memory of subjects with≥6 years of education. Subjects were presented 12 words over 5 trials, followed by delayed recall and recognition trials [3].

3. The Huashan Object Memory Test (HOMT), adapted from the Fuld Object Memory Evaluation [4], was used to measure immediate and delayed memory for subjects with <6 years of education. The test material consists of 12 common objects familiar to Chinese people. Subjects were presented with 12 words to learn over 5 trials, followed by delayed recall and recognition trials.

4. The Modified Common Objects Sorting Test (COST), adapted from the Object Sorting Test [5], was used to measure language. The test material consists of pictures of 42 common objects familiar to Chinese people. Subjects were first required to name each object in the picture. Then subjects were asked to sort all the objects into 7 different groups. Subjects were then asked, “Why do all these belong together?”

5. The Stick Test, adapted from the Stick Construction Test [5], was used as a measure of spatial construction function. This 10-item test was first administered as a copying task. Subjects were given 4 wooden sticks and asked to copy the examiner’s model exactly. Subjects were asked to recall and construct the previous pattern after copying the current one. After the 10 designs were copied, the reversal condition was administered in which subjects were asked to construct the reverse pattern of the examiner’s model.

6. Trail Making Tests (TMT) A and B, adapted from a subtest of the Halstead–Reitan neuropsychological battery [6], were used for subjects with ≥6 years of education. Subjects were required to connect 25 consecutive targets with numbers inside squares or circles on a sheet of paper. There were 2 parts of the test: A, in which the targets were all numbers, and subjects needed to connect them in sequential order (1, 2, 3, etc.); and B, in which subjects were asked to connect numbers in sequential order with the alternation of square and circle (“1” in a square, “2” in a circle, etc.). This test measures attention function.

7. The Renminbi (official currency of China) Test, translated from the EURO Test [7], was used to measure attention function in subjects with <6 years of education. After assessing the subject’s knowledge of different coins and bills, the subject performed 5 arithmetical tasks of increasing difficulty with 11 coins (counting, making change, adding, dividing by 2, and dividing by 3). After a distraction task, the subject was asked to recall the number and type of coins used before, and the total amount of money involved.

8. The Conflicting Instructions Task (Go/No-Go Task), adapted from part of the Frontal Assessment Battery [8], was used to measure executive function. First, subjects were asked to tap fingers following the conflicting instructions (sensitivity to interference): “tap twice when I tap once,” and “tap once when I tap twice.” Then subjects tapped fingers according to the series of “1-1-2-1-2-2-2-1-1-2/-1-2-2-1-1-2-1-2-2-1,” when performed by the examiner. In addition, subjects were asked to do

"Go/No-Go" (inhibitory control): "tap once when I tap once," and "do not tap when I tap twice." Subjects tapped fingers according to the series of "2-1-2-1-1-2-2-1-1-2/-1-2-1-2-2-1-1-2-2-1," performed by the examiner.

REFERENCES

1. Fazekas F, Chawluk JB, Alavi A, Hurtig HI, Zimmerman RA. MR signal abnormalities at 1.5 T in Alzheimer's dementia and normal aging. AJR Am J Roentgenol. 1987; 149:351–56. https://doi.org/10.2214/ajr.149.2.351 PMID:3496763

2. Zhang MY, Katzman R, Salmon D, Jin H, Cai GJ, Wang ZY, Qu GY, Grant I, Yu E, Levy P, Klauber MR, Liu WT. The prevalence of dementia and Alzheimer's disease in Shanghai, China: impact of age, gender, and education. Ann Neurol. 1990; 27:428–37. https://doi.org/10.1002/ana.410270412 PMID:2353798

3. Guo Q, Zhao Q, Chen M, Ding D, Hong Z. A comparison study of mild cognitive impairment with 3 memory tests among Chinese individuals. Alzheimer Dis Assoc Disord. 2009; 23:253–59. https://doi.org/10.1097/WAD.0b013e3181999e92 PMID:19812468

4. Rideaux T, Beaudreau SA, Fernandez S, O'Hara R. Utility of the abbreviated Fuld Object Memory Evaluation and MMSE for detection of dementia and cognitive impairment not dementia in diverse ethnic groups. J Alzheimers Dis. 2012; 31:371–86. https://doi.org/10.3233/JAD-2012-112180 PMID:22555374

5. Lezak MD, Howieson DB, Loring DW, Fischer JS. Neuropsychological assessment. USA: Oxford University Press; 2004.

6. Lu J, Guo Q, Hong Z. Trail making test used by Chinese elderly patients with mild cognitive impairment and mild Alzheimer'dementia. Chin J Clin Psychol. 2006; 14:118.

7. Carnero-Pardo C, Gurpegui M, Sanchez-Cantalejo E, Frank A, Mola S, Barquero MS, Montoro-Rios MT, Trans EG, and Trans-EUROTEST Group. Diagnostic accuracy of the Eurotest for dementia: a naturalistic, multicenter phase II study. BMC Neurol. 2006; 6:15. https://doi.org/10.1186/1471-2377-6-15 PMID:16606455

8. Dubois B, Slachevsky A, Litvan I, Pillon B. The FAB: a Frontal Assessment Battery at bedside. Neurology. 2000; 55:1621–26. https://doi.org/10.1212/WNL.55.11.1621 PMID:11113214
