# Supplementary material for: White matter hyperintensities associated with progression of cerebral small vessel disease: a 7-year Chinese urban community study
Source: Aging (Albany NY). 2020 May 10;12(9):8506–22. doi: 10.18632/aging.103154 (PMC7244059; doi:10.18632/aging.103154)
Supplement: Supplementary Tables [file aging-12-103154-s003.pdf]

SUPPLEMENTARY TABLES

Supplementary Table 1. Baseline characteristics of all individuals (n=350).

	Total (n=350)	Followed (n=191)	Not followed (n=131)	Death (n=28)	P
Demographics					
Age, y, median (IQR)	69.0(63.3 to 73.6)	68.1(63 to 72.6)	70(63.9 to 74.6)	71.9(66.3 to 74.9)	**0.013**
Sex, male, n (%)	159(45.4)	83(43.5)	61(46.6)	15(53.6)	0.572
Education, y, median (IQR)	12(9 to 14)	12(9 to 15)	12(9 to 14)	12(4.5 to 12)	0.088
Vascular risk factors					
Body mass index, kg/m^2, median (IQR)	24.6(22.4 to 27.2)	24.5(21.9 to 27.3)	25.1(22.9 to 27.3)	23.9(22.4 to 26.4)	0.457
ApoE ε4 carriers, n(%)	48(14.0)	26(14.0)	18(13.8)	4(14.3)	0.998
Current smoking, n (%)	44(12.6)	21(11.0)	15(11.5)	8(28.6)	**0.029**
Hypertension, n (%)	186(53.1)	90(47.1)	78(59.5)	18(64.3)	**0.042**
Diabetes, n (%)	47(13.4)	20(10.5)	21(16.0)	6(21.4)	0.154
Hyperlipidemia, n (%)	133(38.0)	74(38.7)	50(38.2)	9(32.1)	0.797
Cardiogenic disease, n (%)	41(11.7)	19(9.9)	16(12.0)	6(21.4)	0.206
Medication use, n (%)					
Antihypertensive	172(49.1)	94(49.2)	61(46.6)	17(60.7)	0.397
Antidiabetic	41(11.7)	23(12.0)	12(9.2)	6(21.4)	0.182
Lipid lowering	18(5.1)	14(7.3)	3(2.3)	1(3.6)	0.120
Antiplatelet / anticoagulation	69(19.7)	48(25.1)	20(15.3)	1(3.6)	**0.006**
CSVD markers					
WMH volume, %, median (IQR)	0.38(0.18 to 0.78)	0.29(0.15 to 0.59)	0.46(0.22 to 0.93)	0.93(0.43 to 1.43)	**<0.001**
Lacunes, n (%)	56(16.0)	22(11.5)	29(22.1)	5(17.9)	**0.024**
CMBs, n (%)[a]	44(13.8)	16(10.1)	23(17.6)	5(17.9)	0.301
Extensive ePVS, n (%)	52(14.9)	23(12.0)	24(18.3)	5(17.9)	0.267
SVD score≥2, n(%)[a]	56(17.7)	17(10.7)	33(25.4)	6(21.4)	**0.004**
Cognition, median (IQR)					
MMSE	29(28 to 30)	29(28 to 30)	29(27 to 30)	29.5(26.5 to 30)	**0.034**
Memory	0.06(-0.66 to 0.70)	0.31(-0.36 to 0.88)	-0.29(-0.88 to 0.50)	-0.09(-0.84 to 0.42)	**<0.001**
Language	0.25(-0.07 to 0.47)	0.36(-0.07 to 0.57)	0.14(-0.29 to 0.47)	-0.07(-0.72 to 0.31)	**0.001**
Spatial construction	-0.13(-0.61 to 0.59)	0.11(-0.61 to 0.83)	-0.13(-0.85 to 0.59)	-0.61(-1.33 to 0.35)	**<0.001**
Attention	-0.18(-0.80 to 0.57)	-0.02(-0.54 to 0.60)	-0.42(-1.06 to 0.14)	-0.60(-1.14 to 0.04)	**<0.001**
Executive function	0.07(-0.09 to 0.23)	0.07(-0.09 to 0.23)	0.07(-0.09 to 0.23)	-0.01(-0.25 to 0.23)	0.344

[a] For ratings of CMBs, 32 participants were additionally excluded based on missing T2*-GRE at baseline.
Abbreviations: WMH = white matter hyperintensities; CMBs = cerebral microbleeds; ePVS = enlarged perivascular spaces; IQR = interquartile range CSVD = cerebral small vessel disease; SVD = small vessel disease; MMSE = Mini-Mental State Examination.

Supplementary Table 2. Relationships between baseline CSVD markers (semi-quantitative) and progression of CSVD markers.

	Change of WMH (%)	Incident lacunes	Incident CMBs	ePVS Progression
	β (95%CI)	OR (95%CI)	OR (95%CI)	OR (95%CI)
**Model 1**				
WMH Fazekas, per score increase	**0.10(0.05,0.15)**	**2.14(1.20,3.79)**	**1.92(1.29,2.84)**	**1.63(1.10,2.42)**
Lacunes, per No. increase	**0.12(0.02,0.23)**	**2.99(1.35,6.61)**	1.28(0.63,2.59)	2.07(0.93,4.57)
CMBs, per No. increase	0.01(-0.03,0.06)	0.65(0.29,1.50)	1.02(0.71,1.48)	0.88(0.63,1.23)
ePVS, per score increase	0.03(-0.05,0.10)	0.75(0.30,1.89)	0.94(0.55,1.61)	0.22(0.11,0.44)
**Model 2**				
WMH Fazekas, per score increase	**0.11(0.06,0.17)**	**2.15(1.16,3.99)**	**1.84(1.23,2.75)**	**1.55(1.03,2.34)**
Lacunes, per No. increase	0.09(-0.01,0.19)	**3.04(1.30,7.09)**	1.27(0.61,2.66)	2.21(0.97,5.03)
CMBs, per No. increase	0.02(-0.02,0.06)	0.64(0.25,1.62)	1.04(0.72,1.50)	0.87(0.62,1.22)
ePVS, per score increase	0.02(-0.05,0.09)	0.75(0.29,1.95)	0.93(0.54,1.61)	0.22(0.11,0.44)
**Model 3**				
WMH Fazekas, per score increase	**0.11(0.05,0.16)**	**3.06(1.38,6.77)**	**1.81(1.19,2.74)**	**1.64(1.06,2.54)**
Lacunes, per No. increase	**0.11(0.01,0.22)**	2.39(0.85,6.70)	1.24(0.57,2.73)	2.01(0.83,4.87)
CMBs, per No. increase	0.02(-0.02,0.06)	0.73(0.34,1.58)	1.02(0.72,1.44)	0.90(0.62,1.30)
ePVS, per score increase	0.03(-0.04,0.10)	0.74(0.20,2.71)	0.79(0.43,1.44)	0.20(0.09,0.42)
**Model 4**				
WMH Fazekas, per score increase	**0.10(0.05,0.15)**	**3.15(1.40,7.12)**	**1.90(1.24,2.92)**	**1.69(1.08,2.65)**
Lacunes, per No. increase	**0.11(0.01,0.22)**	2.38(0.84,6.73)	1.24(0.56,2.75)	2.08(0.85,5.06)
CMBs, per No. increase	0.02(-0.02,0.07)	0.72(0.34,1.53)	0.99(0.69,1.42)	0.93(0.64,1.36)
ePVS, per score increase	0.03 (-0.05,0.10)	0.73(0.20,2.69)	0.78(0.42,1.44)	0.18(0.08,0.38)

Model 1: unadjusted. Model 2: adjusted for baseline age, sex and interval. Model 3: adjusted for baseline age, sex, interval, BMI, ApoE ε4 carrier, current smoking, hypertension, diabetes, hyperlipidemia, cardiogenic disease. Model 4: additionally adjusted for antihypertensive, antidiabetic, lipid lowering, and antiplatelet / anticoagulation medications.
Abbreviations: CSVD = cerebral small vessel disease; WMH = white matter hyperintensities; CMBs = cerebral microbleeds; ePVS = enlarged perivascular spaces.

Supplementary Table 3. Relationships between baseline SVD score and progression of CSVD markers.

	Change of WMH (%)	Incident lacunes	Incident CMBs	ePVS Progression
	**β (95%CI)**	**OR (95%CI)**	**OR (95%CI)**	**OR (95%CI)**
SVD score, per score increase				
Model 1	**0.12(0.06,0.18)**	**1.89(1.13,3.17)**	**2.20(1.44,3.36)**	1.25(0.86,1.81)
Model 2	**0.13(0.07,0.18)**	**1.83(1.06,3.18)**	**2.11(1.36,3.28)**	1.20(0.81,1.77)
Model 3	**0.14(0.07,0.20)**	**3.57(1.53,8.33)**	**2.06(1.28,3.29)**	1.37(0.89,2.09)
Model 4	**0.14(0.08,0.20)**	**3.67(1.57,8.57)**	**2.14(1.32,3.45)**	1.42(0.92,2.19)

Model 1: unadjusted. Model 2: adjusted for baseline age, sex and interval. Model 3: adjusted for baseline age, sex, interval, BMI, ApoE ε4 carrier, current smoking, hypertension, diabetes, hyperlipidemia, cardiogenic disease. Model 4: additionally adjusted for antihypertensive, antidiabetic, lipid lowering, and antiplatelet / anticoagulation medications.
Abbreviations: SVD = small vessel disease; CSVD = cerebral small vessel disease; WMH = white matter hyperintensities; CMBs = cerebral microbleeds; ePVS = enlarged perivascular spaces.

Supplementary Table 4. Relationships between baseline SVD score and change of cognitive function.

	Change of cognitive function											
	MMSE		Memory		Language		Spatial construction		Attention		Executive function	
	β	P	β	P	β	P	β	P	β	P	β	P
SVD score, per score increase	-0.36	0.083	-0.10	0.328	0.01	0.908	0.19	0.171	0.11	0.298	-0.14	0.061

Adjusted for age, sex, interval, education years, ApoE ε4 carrier.
Abbreviations: SVD = small vessel disease; MMSE = Mini-Mental State Examination.

Supplementary Table 5. Relationships between progression of SVD score and change of cognitive function.

	Change of cognitive function											
	MMSE		Memory		Language		Spatial construction		Attention		Executive function	
	β	P	β	P	β	P	β	P	β	P	β	P
SVD score progression, per score increase	-0.12	0.558	0.04	0.630	0.02	0.822	0.27	0.015	0.01	0.953	-0.14	0.053

Adjusted for age, sex, interval, education years, ApoE ε4 carrier.
Abbreviations: SVD = small vessel disease; MMSE = Mini-Mental State Examination.

Supplementary Table 6. Relationships between baseline CSVD markers and change in cognitive diagnosis.

	Change in cognitive diagnosis		
	OR	95%CI	P value
WMH volume, per 1% increase	1.63	0.64,4,16	0.304
Lacunes, per No. increase	1.68	0.80,3.52	0.172
CMBs, per No. increase	0.55	0.16,1.86	0.333
ePVS, per score increase	1.11	0.58,2.14	0.752

Adjusted for age, sex, interval, education years, ApoE ε4 carrier.
Abbreviations: CSVD = cerebral small vessel disease; WMH = white matter hyperintensities; CMBs = cerebral microbleeds; ePVS = enlarged perivascular spaces; OR = odds ratio; CI = confidence interval.

Supplementary Table 7. Relationships between progression of CSVD markers and change in cognitive diagnosis.

	Change in cognitive diagnosis		
	OR	95%CI	P value
Change of WMH volume, per 1% increase	1.47	0.43,5.06	0.543
Incident lacunes, per No. increase	1.03	0.63,1.68	0.912
Incident CMBs, per No. increase	0.90	0.71,1.15	0.408
ePVS progression, per score increase	1.44	0.72,2.91	0.306

Adjusted for age, sex, interval, education years, ApoE ε4 carrier.
Abbreviations: CSVD = cerebral small vessel disease; WMH = white matter hyperintensities; CMBs = cerebral microbleeds; ePVS = enlarged perivascular spaces; OR = odds ratio; CI = confidence interval.
